# The Impact of COVID-19 Infection and Enforced Prolonged Social Isolation on Neuropsychiatric Symptoms in Older Adults With and Without Dementia: A Review

**DOI:** 10.3389/fpsyt.2020.585540

**Published:** 2020-10-22

**Authors:** Riccardo Manca, Matteo De Marco, Annalena Venneri

**Affiliations:** Department of Neuroscience, University of Sheffield, Sheffield, United Kingdom

**Keywords:** Alzheimer's disease, neuropsychiatric, COVID-19, social isolation, delirium, mental health, ageing

## Abstract

**Background:** The sudden and drastic changes due to the Coronavirus Disease 19 (COVID-19) pandemic have impacted people's physical and mental health. Clinically-vulnerable older people are more susceptible to severe effects either directly by the COVID-19 infection or indirectly due to stringent social isolation measures. Social isolation and loneliness negatively impact mental health in older adults and may predispose to cognitive decline. People with cognitive impairments may also be at high risk of worsening cognitive and mental health due to the current pandemic. This review provides a summary of the recent literature on the consequences of COVID-19, due to either viral infection or social isolation, on neuropsychiatric symptoms in older adults with and without dementia.

**Methods:** A search was conducted in PubMed and Web of Science to identify all relevant papers published up to the 7th July 2020. Two independent assessors screened and selected the papers suitable for inclusion. Additional suitable papers not detected by literature search were manually added.

**Results:** Fifteen articles were included: 8 focussed on the psychiatric symptoms caused by the COVID-19 infection and 7 investigated the impact of social isolation on older adults' neuropsychiatric symptoms. Four studies included older adults without dementia and 11 included patients with cognitive impairment mainly due to Alzheimer's disease. All studies found that different neuropsychiatric symptoms emerged and/or worsened in older adults with and without dementia. These changes were observed as the consequence of both COVID-19 infection and of the enforced prolonged conditions of social isolation. Cases were reported of viral infection manifesting with delirium at onset in the absence of other symptoms. Delirium, agitation and apathy were the symptoms most commonly detected, especially in people with dementia.

**Conclusion:** The available evidence suggests that the COVID-19 pandemic has a wide negative impact on the mental well-being of older adults with and without dementia. Viral infection and the consequent social isolation to limit its spreading have a range of neuropsychiatric consequences. Larger and more robustly designed studies are needed to clarify such effects and to assess the long-term implications for the mental health of older adults, and to test possible mitigating strategies.

## Introduction

The current pandemic of *Coronavirus Disease 2019* (COVID-19) has brought abrupt and pervasive changes in our lives that go beyond the infection itself and its consequences on the physical and mental health of those infected. In fact, of equal relevance are the psycho-social consequences generated by the measures put in place worldwide by governments to limit the spreading of COVID-19 and by the traumatic course of events experienced by all those directly involved in this crisis. The biologically-mediated effects of COVID-19 infection have been shown to be multifaceted. Among the many clinical manifestations a variety of neuropsychiatric symptoms (1) and delirium (2) have been observed in patients with severe COVID-19 infection, even in the absence of any other symptoms/signs. Likewise, the psycho-social impact of this pandemic on the mental health of the general population, as well as of frontline workers and people with pre-existing psychiatric conditions, has been extensively documented (3).

Since the beginning of the pandemic, particular concerns have been raised to protect the most clinically vulnerable people in our society, including older adults (i.e., above 60 years old). Analyses carried out using clinical data accumulated over the first half of 2020 and prognostic prediction models clearly show that older adults are particularly vulnerable to COVID-19 infection (4), especially if they are affected by comorbidities such as Alzheimer's disease (AD) (5). The mental well-being of people with dementia who are socially isolating is also considered to be at extremely high risk and a thorough clinical management of this population is regarded as a top priority, especially for those living in care homes, since up to 98% of them present with neuropsychiatric symptoms (6, 7). In fact, a significant association between social isolation and both mental health (8) and levels of cognitive abilities (9) has already been observed in older adults and appears to be mediated by loneliness, i.e., the subjective perception of social isolation. Moreover, greater loneliness has also been found to be significantly associated with reduced brain volume in areas in the left medial temporal lobe involved in memory and harshly affected by AD (10). Consistently, two recent meta-analyses suggested that both poor social engagement/isolation (e.g., living alone, having a limited social network, low frequency of social contact, or inadequate social support) (11) and loneliness (12) may significantly increase the likelihood of developing dementia. Therefore, a suddenly and drastically impoverished social environment may be particularly detrimental to older people, and may contribute to worsen neurological ageing and neurodegeneration-related processes.

**Figure 1:**
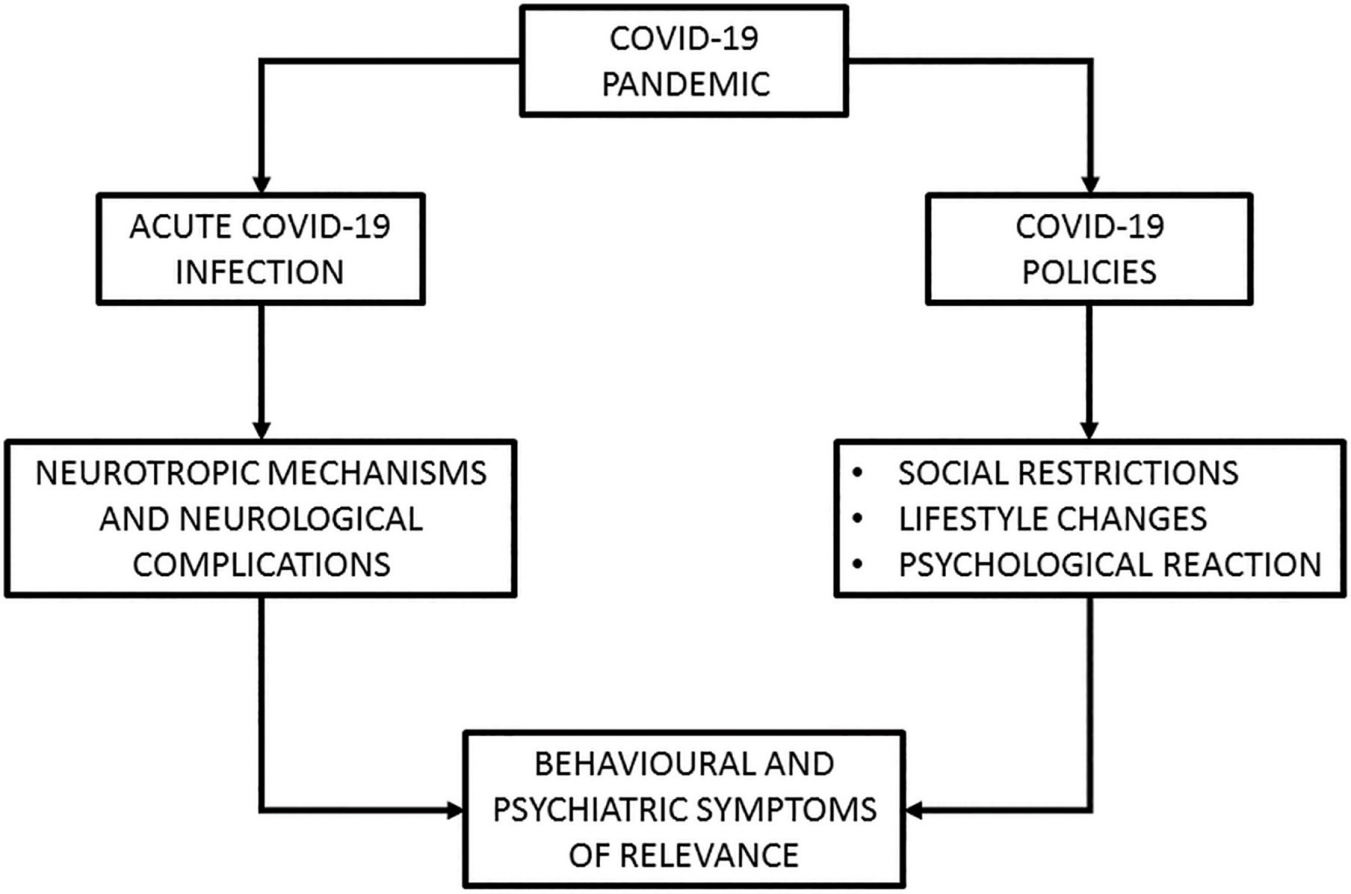
Schematic representation of the theoretical framework whereby the COVID-19 pandemic would be associated to the onset/worsening of behavioural and psychiatric symptoms.

However, many of the questions sparked around the potential detrimental effects of the current pandemic on neuropsychiatric manifestations in older adults still remain unanswered. To address this theoretical gap, the scientific community has been very active in the timely attempt to collect clinical data from the populations of interest. As a result of such hectic efforts, however, the relevant findings are quite scattered at time of writing (July 2020). For this reason, the aim of this review was to summarise the initial wealth of knowledge provided by papers published in the first half of 2020 that reported original data on the effects of the COVID-19 pandemic, both biological (i.e., in individuals who have contracted the virus) and psycho-social (i.e., due to social isolation), on neuropsychiatric symptoms (i.e., behavioural and psychological issues related to the realm of mental health) in older adults with and without dementia. To provide an overview of these two distinct, but inter-connected theoretical aspects, we have included a graphical framework of reference (Figure 1).

## Methods

A systematic literature search was carried out in two online databases, PubMed and Web of Science, to identify equally studies within the remit of medicine and social sciences. A series of keywords regarding the three main factors investigated were used in order to capture all relevant papers: (1) “COVID-19” and “COVID19” for the COVID-19 infection; (2) “dementia,” “mild cognitive impairment,” “neurodegeneration,” “Alzheimer's disease,” “older adults,” “ageing” and “aging” for the populations of interest; (3) “neuropsychiatric,” “psychiatric,” “behavioural,” “behavioral,” “neurobehavioural,” “neurobehavioral” and “delirium” for the specific symptoms. No date-of-publication interval time limits were set for the literature search, but only papers published up to 7th July 2020 (last day of literature search) were eventually included. All publications found were initially screened to identify papers reporting original data, with no restrictions regarding the type of article (e.g., letters and commentaries were included, as long as they presented novel data on the topic of interest). The abstracts of these were reviewed by two independent assessors (MDM and RM) to select all relevant papers to be retained. The exclusion criteria were the following: (1) manuscripts not in English, (2) studies on populations other than those of interest (e.g., children, adolescents, young/middle-aged adults, medical personnel, or general samples of participants not including a distinctive group of older adults), (3) studies focussed on disease and treatment mechanisms, (4) studies investigating other clinical or social/psycho-social aspects of no relevance to this review and (5) non-clinical studies exploring subsidiary topics (e.g., health economics, standards of hygiene or the impact of COVID-19 on insurance companies). A third assessor (AV) helped resolving any disagreement on publications to be included. Additional papers with novel data relevant to this review that were not detected by the literature search but identified through other sources (i.e., references and key journals) were also screened and manually added.

## Results

**Figure 2:**
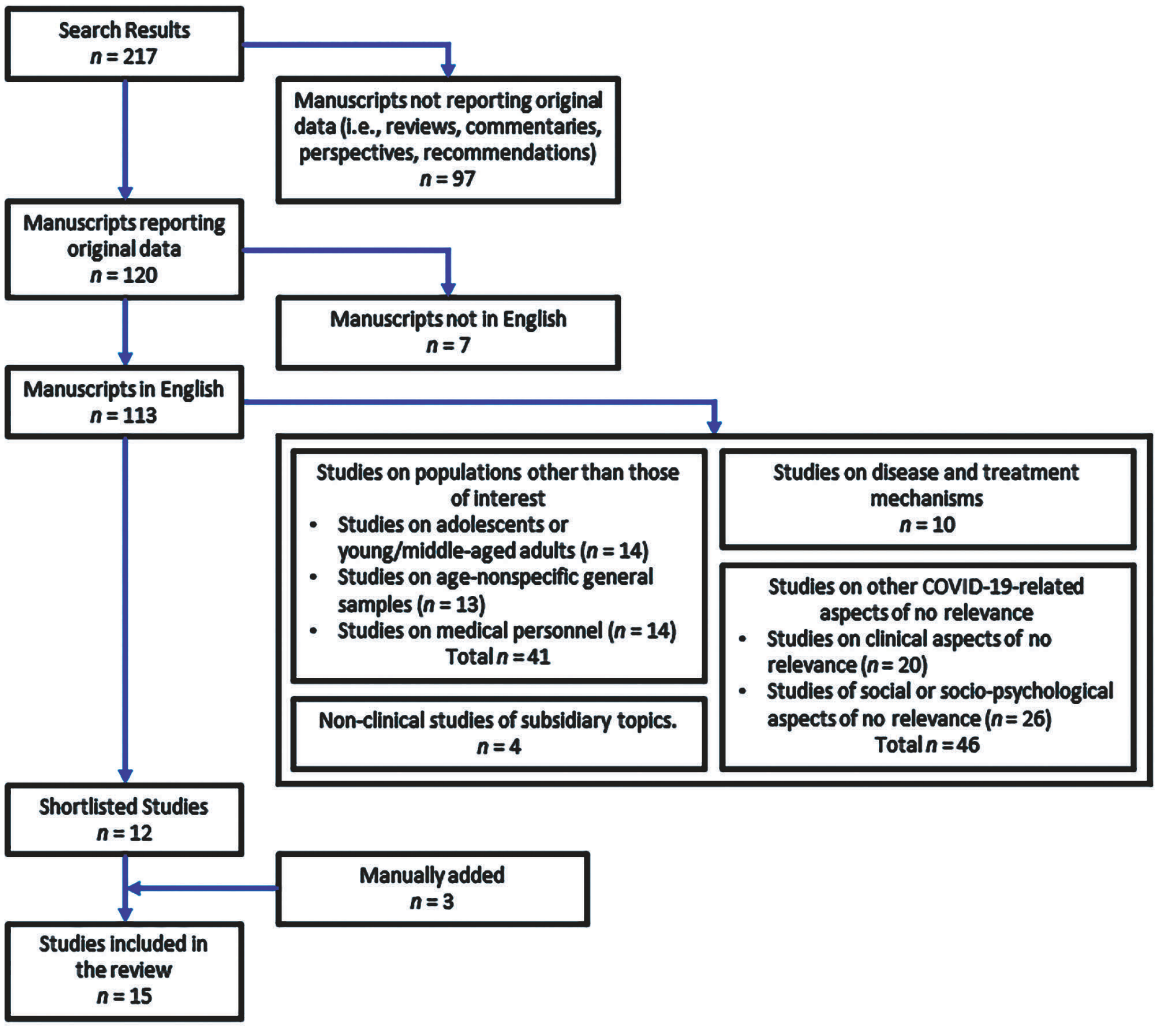
Manuscript selection procedures adopted in this study.

The literature search across the two databases resulted in 344 entries. Of these, 127 were repetitions and were thus discarded. The remaining 217 manuscripts were screened to separate those including original data (i.e., observational studies, case series, single-case descriptions) from those not including original data. This led to 120 manuscripts being retained for further consideration, 7 of which were immediately discarded for not being published in English. It was at this point that each abstract (or, in the case of manuscripts such as letters and commentaries, the entire manuscript) was consulted by the two independent assessors. During the shortlisting process (illustrated in Figure 2), 14 manuscripts described studies carried out on adolescents or young-middle aged adults, and in other 13 manuscripts the age range included younger and older adults without a specific sub-sample of older adults only. Fourteen additional manuscripts were discarded because focussed on the study of medical personnel. Of the 72 remaining manuscripts, 10 focussed on disease mechanisms, 20 addressed clinical aspects of no interest for the current review while another 4 dealt with tangential aspects of the pandemic. Finally, 26 of the remaining manuscripts were discarded because their experimental hypothesis was about social or socio-psychological aspects of no direct relevance for this review. The remaining 12 manuscripts were included in the process of review. Three additional manuscripts of pertinence were found and manually added to this pool of publications, for a total of 15 manuscripts. These are reported in Table 1 together with their main methodological aspects and outcome.

**Table 1:** Characteristics and summary of the results of the studies included.

**Article**	**COVID-19 effects**	**Population diagnosis**	**Methodology**	**Sample size**	**Age (years)**	**Neuropsychiatric symptoms**	**Assessment tools**	**Results**
**INDIVIDUALS WITH ACUTE COVID-19 INFECTION—STUDIES ON OLDER ADULTS WITHOUT DEMENTIA**								
Alkeridy et al. (13)	Effects of infection	Older adults without dementia	Case description	1	73-year-old man	Delirium	Clinical judgment	The patient presented with delirium as onset symptom of COVID-19 infection.
Varatharaj et al. (14)	Effects of infection	Adults without dementia	Multi-centre clinical repository	153 (only 125 with complete assessments)	61–70 (*n* = 23), 71–80 (*n* = 31), 81–90 (*n* = 23), ≥ 91 (*n* = 5)	Altered mental status	Clinical judgment	Altered mental status was observed in 31.2% of the patients with complete assessments: 41% of these had encephalopathy/encephalitis, while 59% met the criteria for different psychiatric diagnoses (91.3% of which were new). The most common disorders were: psychoses, neurocognitive disorders, and affective disorders.
**INDIVIDUALS WITH ACUTE COVID-19 INFECTION—STUDIES INCLUDING OLDER ADULTS WITH DEMENTIA**								
Annweiler et al. (15)	Effects of infection	Older adults with and without MND above 70 years of age	Multi-centre retrospective description of last 10 patients per institution	353 (no MND = 219, MND = 134)	84.7 (±7.0)^*^	Delirium and altered consciousness	Clinical judgment	Older adults with compared to those without MND were more likely to present with delirium, both hypoactive (27.6 vs. 11.4%) and overactive (14.9 vs. 5.5%), and altered consciousness (17.2 vs. 6.4%). Rates of delirium and loss of consciousness were similar between individuals aged 70–80 and over 80.
Beach et al. (16)	Effects of infection	MND (unspecified cause) and DLB	Case series description	3 (an additional case with COVID-19 infection and schizophrenia also included)	70-year-old man, 76-year-old man, and 87-year-old woman	Delirium	Clinical judgment	Two cases of MND, one with behavioural and psychotic problems and one with depression with psychotic features, and one case of DLB presented with delirium and agitation during hospitalisation.
Bianchetti et al. (17)	Effects of infection	Dementia (unspecified cause)	Retrospective analysis of regional acute hospital admissions	627 (no dementia = 545, dementia = 82)	82.6 (± 5.3), IQR 80–86 (dementia)	Behavioural symptoms and delirium	Clinical judgement	At onset: most common symptom in people with dementia was delirium (67%), especially hypoactive (50%); behavioural symptoms were present in 11% of patients
Lovell et al. (18)	Effects of infection	Older adults with and without dementia (unspecified cause)	Retrospective analysis of case series	101 (dementia = 31)	82 (72–89)^†^	Agitation, drowsiness, and delirium	Clinical judgment	At time of referral to palliative care unit, dementia was the third most common comorbidity (30.7%). Overall, 42.5% of patients presented with agitation, 35.6% with drowsiness, and 23.8% with delirium.
Sinvani et al. (19)	Effects of infection	Advanced dementia due to AD	Case series description	1 (other two severe cases of older adults with COVID-19 infection were also included)	76-year-old woman	Behavioural symptoms	Clinical judgment	After a few days of hospitalisation, the patient showed agitation and violent behavioural changes that, however, resolved with personalised care.
Ward et al. (20)	Effects of infection	AD, FTD, VD	Case series description	4 (2 AD; 1 FTD, 1 VD)	83.3 (± 10.2)^*^, 3 women, and 1 man	Altered mental status	Clinical judgment	All cases presented at onset with delirium and agitation. Delirium was particularly severe in 2 cases and associated with loss of appetite and disorientation.
**INDIVIDUALS WITHOUT COVID-19 INFECTION IN CONDITIONS OF INCREASED SOCIAL ISOLATION—STUDIES ON OLDER ADULTS WITHOUT DEMENTIA**								
Emerson et al. (21)	Effects due to social isolation	Older adults without dementia above 60 years of age	Online survey	833	60–70 (*n* = 523), 71+ (*n* = 310)	Overall mental health and stress	Web-based survey, self-reported assessment	No differences in self-rated mental health were found between older adults aged 60–70 and 70+. However, the younger group reported having experienced higher levels of stress than the older group after social isolation enforcement.
Shrira et al. (22)	Effects due to social isolation	Older adults without dementia	Online questionnaire completed by older adults	277	69.58 (± 6.72)^*^, range 60–92	Anxiety, depression, and peritraumatic distress	Web-based GAD-7, PHQ-9, and PDI	Loneliness due to social isolation was positively associated with levels of anxiety, depression and peritraumatic distress, especially among individuals feeling older than their age.
**INDIVIDUALS WITHOUT COVID-19 INFECTION IN CONDITIONS OF INCREASED SOCIAL ISOLATION—STUDIES INCLUDING OLDER ADULTS WITH DEMENTIA**								
Boutoleau-Bretonnière et al. (23)	Effects due to social isolation	Dementia due to probable AD	Telephonic questionnaires administered to a caregiver	38	71.89 (± 8.24)^*^	NPS	NPI-Q	Caregiver-reported worsening of NPS in 26.3% of patients. Duration of confinement correlated with NPI-Q score and caregivers' distress in patients who showed worsening of NPS.
Canevelli et al. (24)	Effects due to social isolation	Dementia, MCI, SCD (unspecified cause)	Telephonic survey administered to patients or caregivers	139 (dementia = 96, MCI/SCD = 43)	80.5 (76–85)^‡^ (dementia); 73 (65.5–77.5)^‡^ (MCI/SCD)	NPS	Patient- and caregiver-reported changes	Overall, NPS improved in only a few patients (2.1% of demented and 7% of MCI/SCD), while NPS worsened in the majority of patients (57.3 and 48.8%, respectively), especially agitation, apathy, depression and irritability.
Fahed et al. (25)	Effects due to social isolation	Dementia due to AD	Case series description	1 (a second case of a patient with narcissistic personality disorder was also included)	83-year-old man	Behavioural symptoms	Clinical judgment	The patient was admitted to an inpatient psychiatric unit during COVID-19 pandemic because of severe agitation. During hospital stay he experienced mood lability, agitation and violent behaviours. All symptoms worsened after he was room isolated because suspected to have COVID-19. All interventions had little or no effect.
Lara et al. (26)	Effects due to social isolation	Dementia due to mild AD and amnestic MCI	Telephonic questionnaires administered to a caregiver	40 (AD = 20, MCI = 20)	77.4 (± 5.25)^*^	NPS	NPI	General worsening of NPI scores was observed after 5 weeks of confinement in agitation, apathy and aberrant motor behaviour symptoms particularly. Changes were similar between patient groups. Apathy and anxiety worsened especially in the MCI group; while apathy, agitation, and aberrant motor behaviours worsened mainly in the AD group.
Padala et al. (27)	Effects due to social isolation	Dementia due to AD	Case description	1	81-year-old man	NPS	NPI	After restrictions were enforced for relatives' visits to people in nursing homes, this patient with AD showed increased depression, anxiety, apathy, irritability, difficulty sleeping, and general restlessness. Symptoms improved after video calls with relative were arranged.

* *mean (± Standard deviation)*.

† *median (Range)*.

‡ *mean (Interquartile range)*.

*AD, Alzheimer's Disease; DLB, Dementia with Lewy Bodies; FTD, Frontotemporal Dementia; GAD-7, 7-item Generalized Anxiety Disorder scale; IQR, Interquartile range; MCI, Mild Cognitive Impairment; MND, Major Neurocognitive disorder; NPI, Neuropsychiatric Inventory; NPI-Q, Neuropsychiatric Inventory Questionnaire; NPS, Neuropsychiatric Symptoms; PDI, 13-item Peritraumatic Distress Inventory; PHQ-9, 9-item Patient Health Questionnaire; SCD, Subjective Cognitive Decline; VD, Vascular Dementia*.

### Individuals With Acute COVID-19 Infection

Eight papers focussed on the neuropsychiatric manifestations of COVID-19 infection, 2 carried out in older adults without dementia (13, 14) and 6 in older adults living with dementia, mostly due to AD aetiology (15–20). Study designs included: one single case (13), three case series (16, 19, 20), two single-centre retrospective analyses of hospital admissions (17, 18) and two multi-centre investigations, one retrospective analysis of COVID-19 cases (15) and one surveillance clinical repository purposely created (14).

#### Studies on Older Adults Without Dementia

Alkeridy et al. (13) described the single case of a 73-year-old man without dementia who resulted positive to testing for COVID-19. The authors observed that this patient presented exclusively with delirium at onset, in the absence of the most common symptoms observed in people infected with COVID-19 (i.e., high fever, dry cough and tiredness), as reported by the outline published by the World Health Organisation (https://www.who.int/health-topics/coronavirus#tab=tab_3). A multi-centre study including 125 patients (most of whom aged 60 or above) with COVID-19 and a complete clinical assessment, found that, at onset, 31.2% presented with, among other symptoms, altered mental status, i.e., acute alteration in personality, behaviour, cognition, or consciousness (14). As many as 59% of these patients met criteria for psychiatric diagnoses, with the great majority being new cases of psychoses, neurocognitive disorders, and affective disorders. In both studies, assessment of neuropsychiatric symptoms was based on a clinician's judgment, and no use of standardised tools was reported.

#### Studies Including Older Adults With Dementia

Three papers described case series reporting a total of 8 patients with dementia due to different underlying conditions: two unspecified and one with dementia with Lewy Bodies (16); 3 cases of AD (19, 20); one case of frontotemporal lobar degeneration and one of vascular dementia (20), respectively. All patients were aged 70 or above, 5 were women and 3 men. In all cases, the neuropsychiatric manifestations of COVID-19 were clinician-reported. At hospitalisation, all patients presented with agitation and 7 out of 8 with delirium. In 2 cases of severe delirium, disorientation and loss of appetite were also reported (20). At least in one case, behavioural disturbance subsided with personalised care (19).

Retrospective investigations of large cohorts of hospitalised patients found that the most common symptoms in those with dementia were delirium, especially in its hypoactive variant, and altered consciousness (15, 17). Similarly, Lovell et al. (18) found that, among the more severe cases of COVID-19 infection admitted to palliative care units, about 30% were people with dementia and many presented with a range of neuropsychiatric symptoms, such as agitation, and delirium.

### Individuals Without COVID-19 Infection in Conditions of Increased Social Isolation

Seven studies focussed on investigating the impact that social isolation due to COVID-19-related restrictions had on neuropsychiatric symptoms of older adults with (23–27) and without dementia (21, 22). Only one single case (27) and one case series (25) were described, while all the other studies used surveys/questionnaires implemented either via online (21, 22) or telephonic (23, 24, 26) administration. The majority of these studies included standardised tools to assess the presence and severity of neuropsychiatric symptoms, mostly the Neuropsychiatric Inventory (NPI) (23, 26, 27).

#### Studies on Older Adults Without Dementia

In a large online survey including 833 healthy older adults (aged ≥ 60) socially isolating during the COVID-19 pandemic, Emerson et al. (21) found no differences in self-rated mental health between older adults aged 60–70 and those aged above 70. However, the younger group reported higher levels of stress than the older group. Shrira et al. (22) observed a significant positive association between loneliness due to social isolation and levels of anxiety, depression, and peri-traumatic distress in older adults. This association was particularly strong for those individuals who felt older than their actual demographic age.

#### Studies Including Older Adults With Dementia

Emergence and worsening of neuropsychiatric symptoms were described in two patients with dementia due to AD after enforcement of social isolation measures. An 83-year-old man was hospitalised due to severe agitation that worsened after he was isolated to his room because suspected to have COVID-19, with little or no relief gained from either pharmacological or non-pharmacological interventions (25). An 81-year-old man, resident in a nursing home, experienced increasing depression, anxiety, apathy, irritability, difficulty sleeping, and general restlessness after his relative's visits had been suspended. All symptoms improved after video calls with his daughter were arranged (27).

In a telephone survey, caregivers of people with cognitive impairment reported mainly worsening of patients' neuropsychiatric symptoms, both when the underlying clinical diagnosis was subjective/mild cognitive impairment (48.8%) and dementia (57.3%), while only a small proportion noticed amelioration of symptoms (24). Greater impacts were especially observed for agitation, apathy, depression, and irritability. Similarly, negative changes in neuropsychiatric symptoms resulting in high NPI scores were reported by two studies: one found symptoms worsening in patients with more compromised cognitive status prior to social isolation and a direct correlation between length of social isolation and both severity of symptoms and caregivers' distress (23); and Lara et al. (26) observed that comparable changes, especially in apathy, occurred in both patients with mild cognitive impairment, and dementia due to AD.

## Discussion

The COVID-19 pandemic has taken the world by storm, inducing an unforeseen course of events that has had a significant impact on our lives. Aside from the medical emergency constituted by the actual viral infection, the diffusion of the virus throughout the world has snowballed into a series of substantial changes to the way we are now compelled to conceive a wide number of aspects of life such as healthcare, employment, financial resources, social interactions, welfare and even simple routine tasks that prior to this pandemic could be taken for granted. This has been a radical turn of events with which societies are coming to terms and, arguably, it will not be an easy task. For this reason, the advent of the pandemic has the potential to act as a major trigger for the onset or exacerbation of certain detrimental psychological traits that in turn may lead to behavioural/psychiatric symptoms of clinical concern. In this context, older people (i.e., older than 60) and people with dementia are among the segments of the population most susceptible to the detrimental effects of COVID-19. On one hand they are clinically vulnerable to the viral infection, on the other hand they are at risk of suffering from the negative consequences of reduced social interactions (Figure 1).

To shed light on this issue, in this review we searched the scientific literature in the attempt of putting together research findings and case descriptions on the topic published over the first half of 2020, focussing on behavioural and psychiatric symptoms, but giving equal emphasis to both “mechanistic” and “reactive” avenues of interference with normal psychological well-being in people undergoing normal or neurodegenerative processes of ageing.

### Neuropsychiatric Symptoms in People Who Have Contracted COVID-19

Societies regularly see annual waves of viral infections during the colder part of the year [e.g., Vestergaard et al. (28)]. While yearly influenza presents itself as a serious yet, in a sense, “canonical” respiratory family of viruses, it has been long established that influenza-associated hospital admissions might present with mental disturbances of psychotic nature (29). Delirium, in particular, is often seen in clinical settings in concomitance with acute hospitalisation and infection. Likewise, a non-negligible amount of clinical evidence has been collected during the current emergency that suggests that COVID-19 may also affect the central nervous system to a significant extent. The evidence we have reviewed in the current manuscript is limited to a small number of studies that converge towards delirium being the most common behavioural symptom recorded at the peak of the infection, and even at onset, in the absence of any other symptoms (13), especially in patients with dementia (17). A substantial proportion of infected patients also experienced mental health problems sufficiently severe to meet criteria for a variety of new psychiatric diagnoses, as well as neurocognitive syndromes, these latter potentially unveiling ongoing latent neurodegenerative processes (14). Particularly affected were people with dementia, who presented often with agitation (16, 18–20) and altered consciousness (15, 18). It is important to remark that these findings were collected in clinical environments that, during the acute phase of the crisis, hosted exclusively severe cases in need of hospitalisation. As a consequence, it still remains undetermined whether milder infections may mechanistically lead to the presence of these or other psychiatric symptoms. Transient agitation in the acute care setting may occur even in an individual who does not have a diagnosis of cognitive impairment or psychiatric disorders, and might be due to a concatenation of neurological and biochemical factors, including an underlying infection, hypoxia, and medication side effects (30). Delirium, instead, is a state of confusion in which a sudden decline in attentional levels and cognitive resources is observed, and is typically seen in hospitalised patients. The occurrence of delirium is determined by a number of predisposing variables (the baseline vulnerability of the individual) as well as precipitating factors introduced during the hospital stay (31). It is well-known that the COVID-19 patients at highest risk of complications are those who show particular frailty (e.g., those who have co-occurring medical conditions). Likewise, major precipitating factors for delirium are “more than three medications added” and “use of bladder catheter” (31), that are a normal occurrence in the hospitalisation of the most severe cases. In summary, it is unfortunate that the frailest who require admission to an intensive care unit are also the more predisposed to developing delirium, and that the routines associated with hospitalisation provide a further hit that might exacerbate their profile. Meanwhile, the current acute neurological and biochemical changes increase the risk of agitation.

Aside from the manifestations recorded in the acute setting, it is possible that behavioural and psychiatric complications might also appear in the long run, in a chronic form (32). Although there still appears to be a paucity of neuropathological research (33), a study carried out on the brains of 18 adults between the ages of 53 and 75 fallen victim to the virus revealed neither CT-informed macrostructural abnormalities, nor microstructural damage ascribable to the virus, but only mild hypoxia-related modifications with, importantly, limited evidence of viral presence in the brain (34). Incoherently with these findings, however, structural magnetic resonance imaging of 30 *in vivo* severe acute cases revealed multifocal subcortical FLAIR and diffusion-weighted signal changes, compatible with oedema, particularly in the mediotemporal lobe, with an aetiological role played by haemorrhagic lesions as well (35). Similarly, a young adult hospitalised because of COVID-19-induced meningitis was described to have hyperintense FLAIR signal in the right hippocampus (36). While these scant pieces of evidence are extremely important to lay the foundations for hypotheses in support of the mechanistic causes of psychiatric and behavioural symptoms in COVID-19, it is too soon to establish a definite theoretical framework and define mechanistic models at the basis of chronic neurological and psychiatric symptoms. Although any mechanistic hypothesis would be, at this stage, speculative, it is of central importance to shed light on the map of regional brain damage caused by the virus, because the topography of network dysfunction may account for the onset of chronic behavioural symptoms. A number of studies has shown that the presence of psychiatric symptoms in patients with AD is associated with alterations of brain circuitry (37–39). If COVID-19 infection damages the neural tissue, it might result into an impoverishment of the neural pathways that support normal psychological functioning and could lead to the onset of cognitive and/or psychiatric dysfunction. Future studies investigating the long-term consequences of COVID-19 on brain function and psychiatric well-being will have the opportunity to address this specific theoretical issue. Moreover, a number of adults who have experienced a particularly severe acute disease might go on developing post-traumatic stress disorder symptoms (1) that would increase the burden on the “reactive” symptomatological profile. In summary, while the presence of delirium and agitation during the acute phase of the infection may be due to a more general neuroinflammatory response, more specific neural mechanisms might underlie the future presence of chronic psychiatric symptoms.

### Neuropsychiatric Symptoms in People in Social Isolation Due to COVID-19 Pandemic

The studies here reviewed also highlight how healthy older adults forced to isolate socially reported high levels of stress (21), anxiety and depression that seem to be particularly associated with loneliness (22). Similarly, worsening or emergence of new neuropsychiatric symptoms was found in a substantial proportion (about 25–60%) of patients with cognitive decline as a result of social isolation (23, 24), although changes in symptom severity were found to be similar between patients with mild cognitive impairment and dementia (26). However, behavioural disturbances observed in some patients either hospitalised (25) or resident in nursing homes (27) appeared to be particularly severe and challenging to manage.

Most of these studies included assessment of neuropsychiatric symptoms by means of standardised tools (e.g., the NPI) compared to those carried out on people affected by COVID-19 that relied on clinical judgment. This is likely to be due to the fact that studies on socially-isolating older adults were conducted remotely, by recruiting people with no COVID-19 infection mostly living at home. Hence, these samples did not necessarily require clinical assessment of acute symptoms. In general, simple and exploratory designs were used, mainly in the form of online/telephone surveys, but control groups and pre-lockdown baseline data were not included, thus preventing definite conclusions on the strength of the recorded changes. Nonetheless, these publications suggest, overall, that conditions of social isolation led to exacerbation or manifestation of a variety of neuropsychiatric symptoms in cognitively healthy older adults (especially stress, mood and anxiety) and those with dementia (mainly agitation and apathy). These reports also provide interesting clues on which social factors might affect both trajectories of cognitive and mental health decline, which appear to be tightly interlinked. Indeed, the presence of neuropsychiatric symptoms is associated with more severe progression of cognitive decline in older adults with (40) and without cognitive impairments (41). Moreover, one of the studies in this review found that patients with AD whose caregivers reported to have experienced worsening of neuropsychiatric symptoms presented with significantly lower global cognitive status before social isolation enforcement (23).

However, a series of variables which can potentially mitigate decline in cognitive and neuropsychiatric symptoms in older adults were not taken into consideration by any of these studies. For instance, the number of people living in the household or the width of the social network in contact with them, e.g., neighbours or online/telephone contacts with friend and relatives. It has long been established that older adults participating in larger social networks appear to show lower rates of dementia (42). Indeed, social network size was found to be positively associated with maintenance of cognitive performance within the normal range over a longer period of time, thus postponing dementia onset independently of APOE status (43). Biomarkers associated with dysfunctional neural processes and AD have also been found to be modulated by patients' social context. Higher levels of serum brain-derived neurotrophic factor (involved, among other functions, in synaptogenesis) have been suggested to play a mediating role between emotional support gained through social engagement and risk of dementia (44). Moreover, patients with AD and larger social networks appeared to retain better cognitive performance even in the presence of high levels of AD pathology, i.e., load of brain amyloid plaques, assessed *post-mortem* (45).

Although the governmental instructions provided to older adults objectively steer towards increased isolation, the extent to which this translates into a psychological sense of loneliness may vary. In fact, the association between social isolation and mental health decline may be particularly mediated by subjective perceptions: Shankar et al. (46) observed that both loneliness and social isolation are significantly associated with cognitive decline over 4 years among older adults and several studies have suggested that the number of close relationships, poor social engagement/isolation and loneliness may significantly increase the likelihood of developing dementia (11, 12, 47). Similarly, older adults who experience both social isolation and loneliness have been found to report poorer health quality, with worse depressive symptoms and a higher number of comorbidities (8, 48–50). In fact, one study included in this review found that the relationship between loneliness and mental health was stronger in those who reported to feel older than their actual age (22).

These findings lead to the suggestion that higher social engagement and support experienced both before and during lockdown due to the COVID-19 pandemic might have had a protective/mitigating role by contributing to cognitive (51) and affective reserve (52). During the past few months we have witnessed a widespread mobilisation of people volunteering to offer support to the more vulnerable individuals in our societies and contributing to reduce social isolation (53). It is probably due to such fast society-wide changes that many older adults also felt to be part of a common effort to limit the spreading of COVID-19 and, as a consequence, experienced less loneliness despite an initial increase in the first phase of lockdown (54). However, it cannot be ruled out that protracted social isolation and/or loneliness might have also affected a range of biological processes (linked to neural dysfunction) that could have contributed to the manifestation of neuropsychiatric symptoms in older adults (55).

This review has highlighted and summarised preliminary findings available at time of writing on the effects that the current COVID-19 pandemic has on mental health of older adults. All the studies included were published in the past few months in a rapid response to the demand to obtain much needed insights on this dramatic situation. Negative effects of both viral infection and social isolation have been reported in older adults with and without dementia. These must be taken into account in order to overcome the challenges related to the delivery of effective care strategies for people with dementia in the last phases and after the end of this pandemic. Future studies in larger cohorts, with more robust designs and theory-grounded will be needed to gain more knowledge about the short-term and long-term biological and psycho-social effects of the COVID-19 pandemic on mental health of specific vulnerable populations of older adults, e.g., people with non-AD dementias that may present with more severe behavioural problems (56, 57), and to ascertain the biological and psycho-social mechanisms that may explain these findings, as well as the possible risk/protective factors.

## Author's Note

This is a summary of independent research carried out at the NIHR Sheffield Biomedical Research Centre (Translational Neuroscience).

## Author Contributions

AV conceived this study. RM and MD designed this study, carried out the literature search, selected the papers for inclusion, summarised the literature findings, and wrote this manuscript. AV reviewed and finalised this manuscript. All authors approved the final version of this manuscript.

## Conflict of Interest

The authors declare that the research was conducted in the absence of any commercial or financial relationships that could be construed as a potential conflict of interest.
